# Dimethyl­ammonium bis­(4-methyl­morpholin-4-ium) tetra­chloridozincate

**DOI:** 10.1107/S1600536811025049

**Published:** 2011-06-30

**Authors:** Yan-wei Zhang, Yan-fei Wang

**Affiliations:** a Department of Chemical & Environmental Engineering, Anyang Institute of Technology, Anyang 455000, People’s Republic of China

## Abstract

The title compound, (C_2_H_8_N)(C_5_H_12_NO)[ZnCl_4_], was synthesized by hydro­thermal reaction of ZnCl_2_ with 4-methyl­morpholine in a dimethyl­formamide solution. The asymmetric unit is composed of half a [ZnCl_4_]^2−^ anion, half a 4-methyl­morpholin-4-ium cation and half a dimethyl­ammonium cation, all located on mirror planes parallel to *ac*. All the amine H atoms are involved in inter­molecular N—H⋯Cl hydrogen bonds, building up an infinite chain parallel to the *c* axis.

## Related literature

For properties of amino compounds, see: Fu *et al.* (2009 ▶); Aminabhavi *et al.* (1986 ▶); Dai & Fu (2008*a* ▶,*b* ▶). 
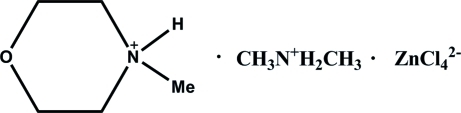


## Experimental

### 

#### Crystal data

(C_2_H_8_N)(C_5_H_12_NO)[ZnCl_4_]
*M* *_r_* = 355.42
Orthorhombic, 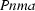

*a* = 20.272 (4) Å
*b* = 10.220 (2) Å
*c* = 7.3727 (15) Å
*V* = 1527.5 (5) Å^3^
*Z* = 4
Mo *K*α radiation
μ = 2.29 mm^−1^
*T* = 298 K
0.30 × 0.05 × 0.05 mm

#### Data collection

Rigaku Mercury2 diffractometer
Absorption correction: multi-scan (*CrystalClear*; Rigaku, 2005 ▶) *T*_min_ = 0.910, *T*_max_ = 1.000
15010 measured reflections
1851 independent reflections
1655 reflections with *I* > 2σ(*I*)
*R*_int_ = 0.031

#### Refinement

*R*[*F* ^2^ > 2σ(*F* ^2^)] = 0.030
*wR*(*F* ^2^) = 0.072
*S* = 1.14
1851 reflections
86 parameters
H atoms treated by a mixture of independent and constrained refinement
Δρ_max_ = 0.38 e Å^−3^
Δρ_min_ = −0.42 e Å^−3^

### 

Data collection: *CrystalClear* (Rigaku, 2005 ▶); cell refinement: *CrystalClear*; data reduction: *CrystalClear*; program(s) used to solve structure: *SHELXS97* (Sheldrick, 2008 ▶); program(s) used to refine structure: *SHELXL97* (Sheldrick, 2008 ▶); molecular graphics: *SHELXTL* (Sheldrick, 2008 ▶); software used to prepare material for publication: *SHELXTL*.

## Supplementary Material

Crystal structure: contains datablock(s) I, global. DOI: 10.1107/S1600536811025049/zk2013sup1.cif

Structure factors: contains datablock(s) I. DOI: 10.1107/S1600536811025049/zk2013Isup2.hkl

Additional supplementary materials:  crystallographic information; 3D view; checkCIF report

## Figures and Tables

**Table 1:** Hydrogen-bond geometry (Å, °)

*D*—H⋯*A*	*D*—H	H⋯*A*	*D*⋯*A*	*D*—H⋯*A*
N1—H1*C*⋯Cl1^i^	0.81 (3)	2.78 (3)	3.435 (2)	139 (1)
N2—H2*D*⋯Cl3^ii^	0.86 (4)	2.42 (4)	3.215 (3)	154 (3)
N1—H1*C*⋯Cl1	0.81 (3)	2.78 (3)	3.435 (2)	139 (1)
N2—H2*C*⋯Cl2	0.85 (4)	2.44 (4)	3.287 (3)	172 (4)

Symmetry codes: (i) 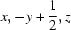; (ii) 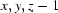.

**Fig. 1.**
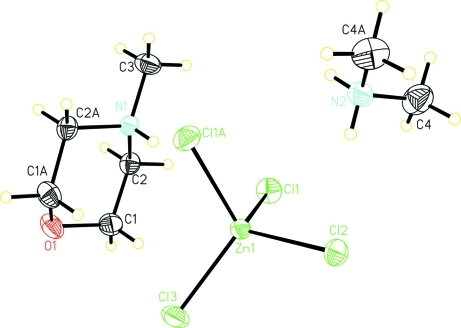
Molecular view of the title compound with the atomic numbering scheme. Displacement ellipsoids are drawn at the 30% probability level.

**Fig. 2.**
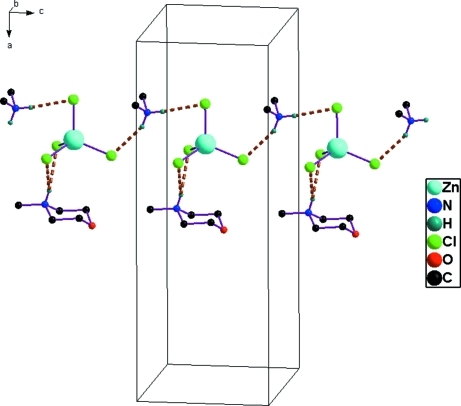
The crystal packing of the title compound viewed along the b axis showing the one-dimensionnal hydrogen bondings chain (dashed line). Hydrogen atoms not involved in hydrogen bonding have been omitted for clarity.

## supplementary crystallographic information

### Comment

The amino derivatives have found wide range of applications in material science, such as magnetic, fluorescent and dielectric behaviors. And there has been an increased interest in the preparation of amino coordination compound (Aminabhavi *et al.,* 1986; Dai & Fu 2008*a*; Dai & Fu 2008*b*; Fu, *et al.* 2009). We report here the crystal structure of the title compound, *Bis-*(4-methylmorpholin-4-ium) (dimethylammonium) tetrachloride Zinc(II).

The asymmetric unit is composed of half ZnCl_4_^2-^ anion, half 4-methylmorpholin-4-ium cation and half dimethylammonium cation (Fig.1). The molecules are located in the *ac* mirror. The geometric parameters of the title compound are in the normal range.

In the crystal structure, all the H atoms of amine groups are involved in intermolecular N—H···Cl hydrogen bonds building up an infinite one-dimensional chain parallel to the *c*-axis (Table 1 and Fig.2).

### Experimental

A mixture of 4-methylmorpholine (0.4 mmol), ZnCl_2_ (0.4 mmol) and DMF/distilled water (10ml,1:1) sealed in a Teflon-lined stainless steel vessel, was maintained at 100 °C. The dimethylamine was generated through the decomposition of DMF. Colorless block crystals suitable for X-ray analysis were obtained after 3 days (yield 31%, based on 4-methylmorpholine). elemental analysis: calcd. C 23.63, H 5.63, N 7.88; found C 23.49, H 5.51, N 7.75.

### Refinement

All H atoms attached to C and N atoms were fixed geometrically and treated as riding with C-H = 0.97 Å(methylene), and C-H = 0.96 Å(methyl) N-H = 0.86 Å, with *U*_iso_(H) = 1.2*U*_eq_(methylene or N) and *U*_iso_(H) = 1.5*U*_eq_(methyl).

### Crystal data

**Table:**

(C_2_H_8_N)(C_5_H_12_NO)[ZnCl_4_]	*F*(000) = 728
*M**_r_* = 355.42	*D*_x_ = 1.546 Mg m^−^^3^
Orthorhombic, *P**n**m**a*	Mo *K*α radiation, λ = 0.71073 Å
Hall symbol: -P 2ac 2n	Cell parameters from 1851 reflections
*a* = 20.272 (4) Å	θ = 3.4–27.5°
*b* = 10.220 (2) Å	µ = 2.29 mm^−^^1^
*c* = 7.3727 (15) Å	*T* = 298 K
*V* = 1527.5 (5) Å^3^	Block, colorless
*Z* = 4	0.30 × 0.05 × 0.05 mm

### Data collection

**Table:**

Rigaku Mercury2 diffractometer	1851 independent reflections
Radiation source: fine-focus sealed tube	1655 reflections with *I* > 2σ(*I*)
graphite	*R*_int_ = 0.031
Detector resolution: 13.6612 pixels mm^-1^	θ_max_ = 27.5°, θ_min_ = 3.4°
CCD profile fitting scans	*h* = −26→25
Absorption correction: multi-scan (*CrystalClear*; Rigaku, 2005)	*k* = −13→13
*T*_min_ = 0.910, *T*_max_ = 1.000	*l* = −9→9
15010 measured reflections	

### Refinement

**Table:**

Refinement on *F*^2^	Primary atom site location: structure-invariant direct methods
Least-squares matrix: full	Secondary atom site location: difference Fourier map
*R*[*F*^2^ > 2σ(*F*^2^)] = 0.030	Hydrogen site location: inferred from neighbouring sites
*wR*(*F*^2^) = 0.072	H atoms treated by a mixture of independent and constrained refinement
*S* = 1.14	*w* = 1/[σ^2^(*F*_o_^2^) + (0.0307*P*)^2^ + 0.7198*P*] where *P* = (*F*_o_^2^ + 2*F*_c_^2^)/3
1851 reflections	(Δ/σ)_max_ < 0.001
86 parameters	Δρ_max_ = 0.38 e Å^−^^3^
0 restraints	Δρ_min_ = −0.42 e Å^−^^3^

### Special details

**Table:**

Geometry. All esds (except the esd in the dihedral angle between two l.s. planes) are estimated using the full covariance matrix. The cell esds are taken into account individually in the estimation of esds in distances, angles and torsion angles; correlations between esds in cell parameters are only used when they are defined by crystal symmetry. An approximate (isotropic) treatment of cell esds is used for estimating esds involving l.s. planes.
Refinement. Refinement of F^2^ against ALL reflections. The weighted R-factor wR and goodness of fit S are based on F^2^, conventional R-factors R are based on F, with F set to zero for negative F^2^. The threshold expression of F^2^ > 2sigma(F^2^) is used only for calculating R-factors(gt) etc. and is not relevant to the choice of reflections for refinement. R-factors based on F^2^ are statistically about twice as large as those based on F, and R- factors based on ALL data will be even larger.

### Fractional atomic coordinates and isotropic or equivalent isotropic displacement parameters (Å^2^)

**Table:**

	*x*	*y*	*z*	*U*_iso_*/*U*_eq_	
Zn1	0.141654 (15)	0.2500	0.85173 (4)	0.03264 (11)	
N1	−0.04000 (12)	0.2500	0.6307 (3)	0.0340 (5)	
H1C	−0.0015 (17)	0.2500	0.663 (4)	0.041*	
Cl2	0.25325 (4)	0.2500	0.84857 (11)	0.0495 (2)	
O1	−0.09518 (12)	0.2500	0.9876 (3)	0.0541 (6)	
Cl3	0.10112 (4)	0.2500	1.13530 (10)	0.0585 (3)	
Cl1	0.10455 (3)	0.07882 (5)	0.68372 (8)	0.04847 (16)	
C2	−0.06975 (11)	0.1302 (2)	0.7135 (3)	0.0402 (5)	
H2A	−0.0472	0.0530	0.6686	0.048*	
H2B	−0.1159	0.1240	0.6795	0.048*	
C1	−0.06386 (13)	0.1363 (2)	0.9169 (3)	0.0507 (6)	
H1A	−0.0839	0.0590	0.9695	0.061*	
H1B	−0.0176	0.1370	0.9506	0.061*	
C3	−0.0450 (2)	0.2500	0.4300 (4)	0.0532 (9)	
H3A	−0.0240	0.3267	0.3821	0.080*	
H3B	−0.0907	0.2500	0.3961	0.080*	
N2	0.22432 (15)	0.2500	0.4099 (4)	0.0492 (7)	
H2C	0.2277 (19)	0.2500	0.525 (5)	0.059*	
H2D	0.184 (2)	0.2500	0.370 (5)	0.059*	
C4	0.25540 (16)	0.1297 (3)	0.3477 (4)	0.0701 (8)	
H4A	0.2331	0.0559	0.3998	0.105*	
H4B	0.3008	0.1289	0.3844	0.105*	
H4C	0.2528	0.1249	0.2178	0.105*	

### Atomic displacement parameters (Å^2^)

**Table:**

	*U*^11^	*U*^22^	*U*^33^	*U*^12^	*U*^13^	*U*^23^
Zn1	0.02909 (17)	0.0403 (2)	0.02851 (17)	0.000	−0.00062 (12)	0.000
N1	0.0280 (11)	0.0368 (13)	0.0371 (13)	0.000	0.0001 (10)	0.000
Cl2	0.0281 (4)	0.0729 (6)	0.0476 (4)	0.000	−0.0013 (3)	0.000
O1	0.0637 (15)	0.0544 (14)	0.0441 (13)	0.000	0.0202 (11)	0.000
Cl3	0.0404 (4)	0.1060 (8)	0.0291 (4)	0.000	0.0030 (3)	0.000
Cl1	0.0547 (3)	0.0400 (3)	0.0508 (3)	−0.0067 (2)	−0.0042 (2)	−0.0089 (2)
C2	0.0421 (11)	0.0304 (10)	0.0479 (12)	−0.0014 (9)	0.0017 (9)	0.0011 (9)
C1	0.0591 (14)	0.0453 (13)	0.0476 (12)	0.0025 (11)	0.0076 (11)	0.0104 (11)
C3	0.072 (2)	0.054 (2)	0.0340 (16)	0.000	0.0016 (16)	0.000
N2	0.0449 (15)	0.0621 (18)	0.0405 (14)	0.000	0.0003 (13)	0.000
C4	0.0778 (19)	0.0614 (18)	0.0712 (18)	0.0134 (15)	−0.0120 (15)	−0.0059 (15)

### Geometric parameters (Å, °)

**Table:**

Zn1—Cl3	2.2464 (9)	C2—H2B	0.9700
Zn1—Cl2	2.2625 (9)	C1—H1A	0.9700
Zn1—Cl1	2.2717 (6)	C1—H1B	0.9700
Zn1—Cl1^i^	2.2717 (6)	C3—H3A	0.9597
N1—C3	1.483 (4)	C3—H3B	0.9597
N1—C2	1.495 (2)	N2—C4	1.456 (3)
N1—C2^i^	1.495 (2)	N2—C4^i^	1.456 (3)
N1—H1C	0.81 (3)	N2—H2C	0.85 (4)
O1—C1	1.423 (3)	N2—H2D	0.86 (4)
O1—C1^i^	1.423 (3)	C4—H4A	0.9600
C2—C1	1.505 (3)	C4—H4B	0.9600
C2—H2A	0.9700	C4—H4C	0.9600
			
Cl3—Zn1—Cl2	112.05 (3)	O1—C1—H1A	109.4
Cl3—Zn1—Cl1	112.73 (2)	C2—C1—H1A	109.4
Cl2—Zn1—Cl1	108.99 (2)	O1—C1—H1B	109.4
Cl3—Zn1—Cl1^i^	112.73 (2)	C2—C1—H1B	109.4
Cl2—Zn1—Cl1^i^	108.99 (2)	H1A—C1—H1B	108.0
Cl1—Zn1—Cl1^i^	100.72 (4)	N1—C3—H3A	109.7
C3—N1—C2	112.33 (16)	N1—C3—H3B	109.1
C3—N1—C2^i^	112.33 (16)	H3A—C3—H3B	109.5
C2—N1—C2^i^	109.9 (2)	C4—N2—C4^i^	115.3 (3)
C3—N1—H1C	111 (2)	C4—N2—H2C	106.2 (13)
C2—N1—H1C	105.6 (12)	C4^i^—N2—H2C	106.2 (13)
C2^i^—N1—H1C	105.6 (12)	C4—N2—H2D	107.4 (12)
C1—O1—C1^i^	109.5 (2)	C4^i^—N2—H2D	107.4 (12)
N1—C2—C1	109.96 (19)	H2C—N2—H2D	115 (4)
N1—C2—H2A	109.7	N2—C4—H4A	109.5
C1—C2—H2A	109.7	N2—C4—H4B	109.5
N1—C2—H2B	109.7	H4A—C4—H4B	109.5
C1—C2—H2B	109.7	N2—C4—H4C	109.5
H2A—C2—H2B	108.2	H4A—C4—H4C	109.5
O1—C1—C2	111.3 (2)	H4B—C4—H4C	109.5
			
C3—N1—C2—C1	−179.4 (2)	C1^i^—O1—C1—C2	−61.7 (3)
C2^i^—N1—C2—C1	−53.6 (3)	N1—C2—C1—O1	58.1 (3)

Symmetry codes: (i) *x*, −*y*+1/2, *z*.

### Hydrogen-bond geometry (Å, °)

**Table:**

*D*—H···*A*	*D*—H	H···*A*	*D*···*A*	*D*—H···*A*
N1—H1C···Cl1^i^	0.81 (3)	2.78 (3)	3.435 (2)	139.(1)
N2—H2D···Cl3^ii^	0.86 (4)	2.42 (4)	3.215 (3)	154 (3)
N1—H1C···Cl1	0.81 (3)	2.78 (3)	3.435 (2)	139.(1)
N2—H2C···Cl2	0.85 (4)	2.44 (4)	3.287 (3)	172 (4)

Symmetry codes: (i) *x*, −*y*+1/2, *z*; (ii) *x*, *y*, *z*−1.

## References

[bb1] Aminabhavi, T. M., Biradar, N. S. & Patil, S. B. (1986). *Inorg. Chim. Acta*, **125**, 125–128.

[bb2] Dai, W. & Fu, D.-W. (2008*a*). *Acta Cryst.* E**64**, m1016. 10.1107/S1600536808019168 PMC2961939 21203009

[bb3] Dai, W. & Fu, D.-W. (2008*b*). *Acta Cryst.* E**64**, m1017. 10.1107/S1600536808006454 PMC2961940 21203010

[bb4] Fu, D.-W., Ge, J.-Z., Dai, J., Ye, H.-Y. & Qu, Z.-R. (2009). *Inorg. Chem. Commun.* **12**, 994–997.

[bb5] Rigaku (2005). *CrystalClear* Rigaku Corporation, Tokyo, Japan.

[bb6] Sheldrick, G. M. (2008). *Acta Cryst.* A**64**, 112–122. 10.1107/S0108767307043930 18156677

